# Electrophysiological Evaluation of Pacific Oyster (*Crassostrea gigas*) Sensitivity to Saxitoxin and Tetrodotoxin

**DOI:** 10.3390/md19070380

**Published:** 2021-06-30

**Authors:** Floriane Boullot, Caroline Fabioux, Hélène Hégaret, Pierre Boudry, Philippe Soudant, Evelyne Benoit

**Affiliations:** 1 Service d’Ingénierie Moléculaire Pour la Santé (SIMoS), Département Médicaments et Technologies Pour la Santé (DMTS), Institut des Sciences du Vivant Frédéric Joliot, Université Paris-Saclay, CEA, ERL CNRS/CEA 9004, 91191 Gif-sur-Yvette, France; floriane.boullot@gmail.com; 2 Laboratoire des Sciences de l’Environnement Marin (LEMAR), Institut Universitaire Européen de la Mer, Université de Bretagne Occidentale, UMR 6539 CNRS/UBO/IRD/Ifremer, 29280 Plouzané, France; caroline.fabioux@univ-brest.fr (C.F.); helene.hegaret@univ-brest.fr (H.H.); philippe.soudant@univ-brest.fr (P.S.); 3 Centre Ifremer Bretagne, Ifremer, UMR 6539 (LEMAR) CNRS/UBO/IRD/Ifremer, ZI de la Pointe du Diable, CS 10070, 29280 Plouzané, France; pierre.boudry@ifremer.fr

**Keywords:** *Crassostrea gigas*, compound nerve action potential, *Alexandrium minutum*, paralytic shellfish toxins

## Abstract

Pacific oysters (*Crassostrea gigas*) may bio-accumulate high levels of paralytic shellfish toxins (PST) during harmful algal blooms of the genus *Alexandrium*. These blooms regularly occur in coastal waters, affecting oyster health and marketability. The aim of our study was to analyse the PST-sensitivity of nerves of Pacific oysters in relation with toxin bio-accumulation. The results show that *C. gigas* nerves have micromolar range of saxitoxin (STX) sensitivity, thus providing intermediate STX sensitivity compared to other bivalve species. However, theses nerves were much less sensitive to tetrodotoxin. The STX-sensitivity of compound nerve action potential (CNAP) recorded from oysters experimentally fed with *Alexandrium minutum* (toxic-alga-exposed oysters), or *Tisochrysis lutea*, a non-toxic microalga (control oysters), revealed that oysters could be separated into STX-resistant and STX-sensitive categories, regardless of the diet. Moreover, the percentage of toxin-sensitive nerves was lower, and the STX concentration necessary to inhibit 50% of CNAP higher, in recently toxic-alga-exposed oysters than in control bivalves. However, no obvious correlation was observed between nerve sensitivity to STX and the STX content in oyster digestive glands. None of the nerves isolated from wild and farmed oysters was detected to be sensitive to tetrodotoxin. In conclusion, this study highlights the good potential of cerebrovisceral nerves of Pacific oysters for electrophysiological and pharmacological studies. In addition, this study shows, for the first time, that *C. gigas* nerves have micromolar range of STX sensitivity. The STX sensitivity decreases, at least temporary, upon recent oyster exposure to dinoflagellates producing PST under natural, but not experimental environment.

## 1. Introduction

Harmful Algal blooms (HAB) are worldwide phenomena that have increased in frequency during the past few decades [1,2]. Notably, French coasts are regularly affected by HAB. Some HAB are attributable to dinoflagellates of the *Alexandrium* genus, mainly *A. minutum* and *A. catenella*, which produce paralytic shellfish toxins (PST) [3,4]. PST constitute a broad group of natural neurotoxic alkaloids composed of saxitoxin (STX) and analogues [5] which, similarly to tetrodotoxin (TTX), block voltage-gated sodium (Na_v_) channels and thus inhibit action potentials in many excitable cells [6].

When HAB occur, filter-feeders, such as bivalve molluscs that feed on phytoplankton, may bio-accumulate large amounts of PST. Consumption of PST-contaminated shellfish, the main vectors of PST to humans, leads to paralytic shellfish poisoning, one of the highest worldwide threats to human health among phycotoxin human poisoning syndromes [2,7]. In addition, this significant human health risk affects the economy linked to shellfish harvesting or aquaculture because of closures and sale prohibitions [8]. In Europe, PST load in shellfish is subjected to regulation defined by the European Union (CE Regulation No. 854/2004) which prohibits the harvesting and sale of shellfish having more than 800 μg equivalent STX per kg (STX-eq.kg^−1^) of fresh meat.

The bio-accumulation of PST in bivalve molluscs was reported to be partly dependent upon the sensitivity of the species to these neurotoxins, linked to nerve sensitivity [9,10]. Indeed, PST bind to Na_v_ channels inducing a neuronal dysfunction. The most striking example comes from the case of American populations of *Mya arenaria* softshell clams. The populations of clams regularly exposed to PST-producer *Alexandrium* spp. accumulate more PST and have lower nerve sensitivity to STX than clams from populations free of blooms [9]. This difference in sensitivity to PST results from the existence of two Na_v_ genotypes, distinguishing PST-sensitive and PST-resistant clams, probably resulting from a long time selection process of resistant genotypes in populations regularly exposed to *Alexandrium* spp. [9]. In French Pacific oyster populations experimentally or naturally exposed to a toxic strain of *A. minutum,* a very high inter-individual variability (up to 450 fold difference) in PST bio-accumulation was observed without shellfish mortality [11,12]. However, the biological bases of this variability remained to be investigated.

Several studies were undertaken to investigate the effects of PST and TTX on Na_v_ channels of bivalve molluscs, notably focusing on nerve cells [10,13]. Comparing different species of bivalves for the sensitivity of their nerves to STX and TTX, Twarog (1974) demonstrated that, in general, toxin-resistant species bio-accumulate higher levels of PST [14]. Similarly, some bivalve species showing a relatively low level of bio-accumulated PST were reported to be highly sensitive to these toxins [10]. However, toxin-sensitivity of *Crassostrea gigas* and its relation with PST bio-accumulation, remained to be investigated.

Thus, the aim of our study was to analyse the STX nerve sensitivities of *C. gigas* in relation to PST bio-accumulation in the digestive gland, the organ accumulating highest PST quantities. In addition, the TTX sensitivity of nerves isolated from wild and farmed oysters was compared because of previously reported similarity between STX and TTX interactions with Na_v_ channels [6].

## 2. Results

### 2.1. Stimulation and Maintaining Conditions of Isolated Oyster Nerves to Obtain Consistent CNAP

**Figure 1:**
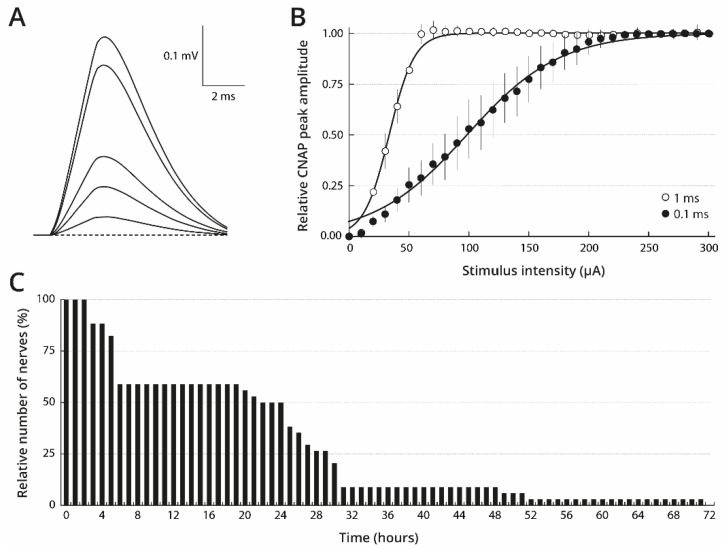
Stimulation and keeping oyster nerve conditions to obtain consistent CNAP. (**A**) Superimposed traces of typical CNAP recorded from the cerebrovisceral nerve stimulated with pulse intensities of 20, 50, 80, 170 and 300 µA and 1-ms duration. (**B**) CNAP peak amplitude (mean ± SE of 22–34 nerves) as function of the intensity of 0.1-ms (●) and 1-ms (○) duration stimulus. For each nerve, the peak amplitude is expressed relatively to its maximal amplitude. The curves correspond to data fits according to the Boltzmann equation with I_50_ = 99 ± 8 µA and S = 39 ± 7 µA^−1^ (●) and I_50_ = 33 ± 3 µA and S = 10 ± 1 µA^−1^ (○). (**C**) Number of nerves able to produce a CNAP in response to electrical stimulation, expressed as the percentage of total number of functional nerves at time zero (*n* = 34), as function of time after their dissection. The nerves were kept in standard physiological solution at 4 °C between recording sessions.

Under our experimental conditions, oyster cerebrovisceral nerves produced monophasic, compound nerve action potentials (CNAP) in response to single electrical stimuli (Figure 1A). The observation of a single form for these monophasic CNAP strongly suggests that this type of nerve is composed of axons having a relatively homogeneous distribution in diameter size. The CNAP peak amplitude was dependent upon stimulus intensity. Hence, in response to stimuli of fixed duration (i.e., 1 ms) and increasing intensities (0–300 µA), the CNAP peak amplitude increased, reached a plateau value, and then remained constant despite further intensity increases (Figure 1B). The 1 ms-stimulus intensity producing a CNAP with 50% of maximal peak amplitude (I_50_) was found to be 33 ± 3 µA (*n* = 22). The CNAP peak amplitude behaved similarly in response to stimuli of decreased duration (i.e., 0.1 ms) with, however, an increased I_50_ value, i.e., 99 ± 8 µA (*n* = 34). The fact that the CNAP peak amplitude attained a plateau indicates that all the axons which could be electrically stimulated, under our experimental conditions, were recruited by stimuli of 0.1–1 ms and 200–300 µA. Under these conditions, the CNAP maximal peak amplitude was found to be 0.422 ± 0.094 mV and to propagate at a velocity of 2.6 ± 0.7 m/s for 34 nerves freshly dissected from wild oysters (collected on October 2015) and kept in standard physiological solution for 15–30 min.

Despite the fact that the nerves were neither bathed nor superfused during the recording periods (less than 5 min duration) (see Materials and Methods), CNAP responses were not modified significantly if the nerves were kept in standard physiological solution for less than 2 h at room temperature, between recordings (*n* = 34). Nerve survival, however, was favoured by storage in standard physiological solution at 4 °C. Under these conditions, 50% of the nerves (*n* = 17) survived for 24 h after dissection (i.e., they could produce a CNAP in response to electrical stimulation), but only 9% (*n* = 3) survived for 48 h, and none were functional at 72 h (Figure 1C).

### 2.2. Basal STX Sensitivity of Isolated Nerves

**Figure 2:**
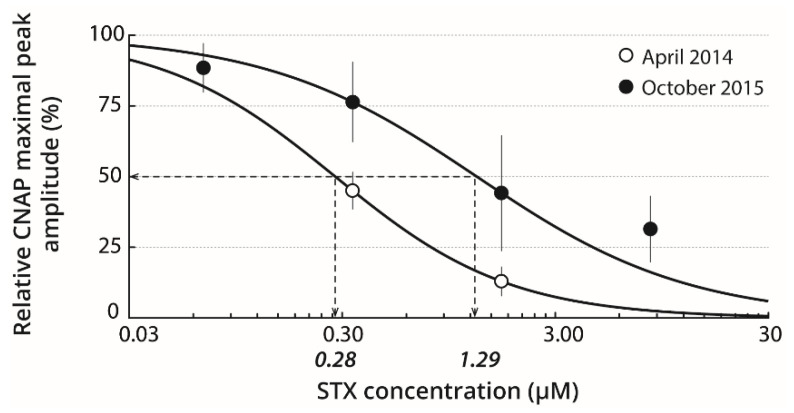
STX sensitivity of nerves isolated from wild oysters. Concentration-response curves of STX effects on the CNAP maximal peak amplitude recorded from wild oysters collected on April 2014 (○) and October 2015 (●). Each value represents the mean ± SE of data obtained from 2 to 9 nerves, and is expressed as percentage of its value obtained in absence of toxin. The curves were calculated from typical sigmoid nonlinear regressions through data points, according to the Hill equation, with IC_50_ values (italic numbers) of 0.28 and 1.29 µM and n_H_ values of 1.06 and 0.87 for nerves isolated from oysters collected on April 2014 and October 2015, respectively.

The STX sensitivity of nerves isolated from wild oysters, collected on April 2014 or October 2015 in the Bay of Brest, was studied by keeping the dissected nerves in standard physiological solution for 15–30 min before a first recording session and then, in a given STX-containing solution for 20–30 min before a second recording session. As illustrated in Figure 2, concentration-response curves of CNAP maximal peak amplitude to STX revealed IC_50_ of 0.28 and 1.29 µM for the nerves isolated from oysters collected during April 2014 and October 2015, respectively. An important inter-individual variability could be observed in mean CNAP of oysters collected in the wild.

The STX-induced blocking effect on CNAP amplitude was reversed by keeping the nerves in standard physiological solution for at least 2 h. Under these conditions, the CNAP maximal peak amplitude, relative to that before pre-treatment of nerves with 8.39 µM STX, was 0.82 ± 0.12 (*n* = 7). In addition, the nerves were pre-treated for 20–30 min with standard physiological solution containing 0.38 mM of hydrochloric acid to verify that STX solvent had, by itself, no detectable effect on the CNAP. The CNAP maximal peak amplitude was 0.97 ± 0.13 (*n* = 11) when expressed relatively to that of nerves before pre-treatment, indicating the absence of solvent effects on CNAP.

### 2.3. STX Sensitivity of Nerves Isolated from Oysters Pre-Exposed to Toxic or Non-Toxic Microalgae

To investigate (i) the effect of *A. minutum* dinoflagellate exposure upon oyster nerve CNAP parameters and (ii) the possible influence of a pre-exposure to *A. minutum* on STX sensitivity, oysters were fed with a toxic strain of *A. minutum* (toxic-alga-exposed oysters) or a non-toxic microalgae (control oysters) just prior to measuring CNAP parameters with or without STX incubation. Keeping dissected nerves in standard physiological solution prior to recording was first ensured to not modify the potential effect of toxic-exposure upon CNAP kinetics and variables. For this purpose, the nerves, once isolated from oysters, were submitted directly to a first recording session and then kept in standard physiological solution for less than 1 min before undergoing a second recording session. Under these conditions, no noticeable difference was detected in the kinetics of CNAP between first and second recordings, i.e., those occurring after having soaked nerves in standard physiological solution, regardless of the microalgae species (toxic or non-toxic) (Supplementary Figure S1A,B). Indeed, none of the six response variables measured to characterise CNAP kinetics was statistically different (Table 1).

In contrast, comparison between nerves isolated from toxic-alga-exposed and control oysters revealed significant differences in CNAP kinetics and variables, following both first and second recordings (Supplementary Figure S1C,D, and Table 1). In particular, the CNAP of toxic-alga-exposed oyster nerves had smaller maximal peak amplitude, increased half-width and time-to-peak, as well as decreased maximal rise and decay slopes, but similar area, when compared with control oyster nerves.

**Figure 3:**
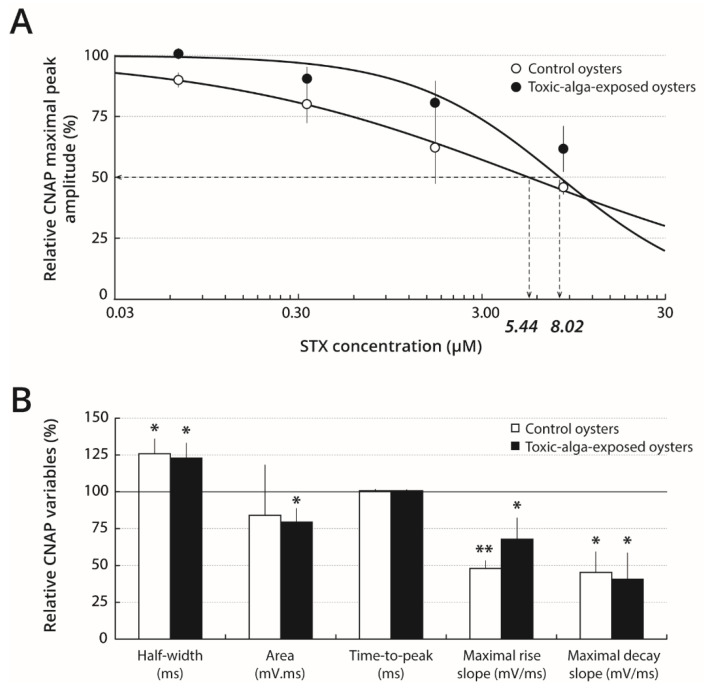
STX effects on relatively sensitive nerves of farmed oysters fed with *A. minutum* (toxic-alga-exposed oysters) or with *Tisochrysis lutea* (control oysters). (**A**) Concentration-response curves of STX effects on the CNAP maximal peak amplitude recorded from toxic-alga-exposed (●) and control (○) oysters. Each value represents the mean ± SE of data obtained from 2–8 nerves, and is expressed as percentage of its value obtained in absence of toxin. The curves were calculated from typical sigmoid nonlinear regressions through data points, according to the Hill equation, with IC_50_ values (italic numbers) of 8.02 and 5.44 µM and n_H_ values of 1.06 and 0.49 for nerves isolated from toxic-alga-exposed and control oysters, respectively. (**B**) Histogram of variables characterising the CNAP kinetics of nerves isolated from toxic-alga-exposed (■) and control (□) oysters and pre-treated with 8.39 µM STX. Each value represents the mean ± SE of data obtained from 6–8 nerves, and is expressed as percentage of its value measured in absence of toxin. * 0.01 < *p* < 0.05 and ** 0.001 < *p* < 0.01, compared with values determined before pre-treatment of nerves with STX.

After pre-treatment of nerves with 0.07–8.39 µM STX, toxic-alga-exposed and control oysters could be separated into two categories according to response to 8.39 µM STX compared with response before pre-treatment (Supplementary Figure S2): the nerves with relative CNAP maximal peak amplitude higher than 80% were called STX-resistant nerves (R-nerves) and, inversely, the nerves with relative CNAP maximal peak amplitude lower than 80% were called STX-sensitive nerves (S-nerves). Under these conditions, 67% of the eighteen nerves isolated from toxic-alga-exposed oysters were qualified as resistant to the toxin, whereas, 33% were qualified as sensitive. In particular, the CNAP maximal peak amplitudes of resistant and sensitive nerves pre-treated with 8.39 µM STX were 96 ± 10% (*n* = 12) and 62 ± 9% (*n* = 6), respectively, of their values determined prior to pre-treatment (Supplementary Figure S3A and Figure 3A). In comparison, only 56% of the eighteen nerves isolated from control oysters were resistant to STX, but 44% of nerves were sensitive to 8.39 µM STX with relative CNAP maximal peak amplitudes of 98 ± 15% (n = 10) and 46 ± 3% (*n* = 8), respectively, of their relative values prior to pre-treatment (Supplementary Figure S3A and Figure 3A). Indeed, the CNAP maximal peak amplitude of resistant nerves of both toxic-alga-exposed and control oysters was not affected by 0.07–8.39 µM STX (Supplementary Figure S3A). However, IC_50_ values determined from concentration-response relationships, *i.e.* the CNAP maximal peak amplitude as a function of STX concentration, were found to be 8.02 and 5.44 µM for sensitive nerves of toxic-alga-exposed and control oysters, respectively (Figure 3A). It thus appears that the relative STX sensitivity of CNAP of toxic-alga-exposed oysters was about 1.5 fold lower than that of control oysters.

The STX-induced blocking effect upon CNAP amplitude of sensitive nerves was reversed by keeping the nerves in standard physiological solution for at least 2 h as, under these conditions, the CNAP maximal peak amplitudes were 93 ± 8% (*n* = 6) and 92 ± 21% (*n* = 7) for toxic-alga-exposed and control oysters, respectively.

The kinetics of CNAP recorded from STX-sensitive and STX-resistant nerves of toxic-alga-exposed and control oysters were analysed by measuring the CNAP variables before and after pre-treatment of nerves with 8.39 µM STX. Comparison of variables of untreated nerves isolated from control oysters revealed that the CNAP of STX-resistant nerves had smaller maximal peak amplitude and increased half-width, as well as decreased maximal rise and decay slopes, but similar time-to-peak and area, when compared with STX-sensitive nerves. In contrast, no significant difference was detected in the kinetics of CNAP between untreated STX-resistant and STX-sensitive nerves isolated from toxic-alga-exposed oysters (Table 2). After pre-treatment of nerves with 8.39 µM STX, the CNAP half-width, time-to-peak and area, as well as maximal rise and decay slopes of STX-resistant nerves isolated from toxic-alga-exposed and control oysters, were not statistically different from their respective values determined before pre-treatment of nerves (Supplementary Figure S3B). In contrast, the CNAP of STX-sensitive nerves isolated from toxic-alga-exposed and control oysters, and pre-treated with 8.39 µM STX, presented an increased half-width and decreased maximal rise and decay slopes, but similar time-to-peak and, at least for control oysters, area, when compared with respective values determined before pre-treatment of nerves (Figure 3B). Taken altogether, these results suggest that the “resistance” characteristic of nerves to STX was consistently associated with CNAP having relatively small maximal peak amplitude, high half-width, and low maximal rise and decay slopes.

**Figure 4:**
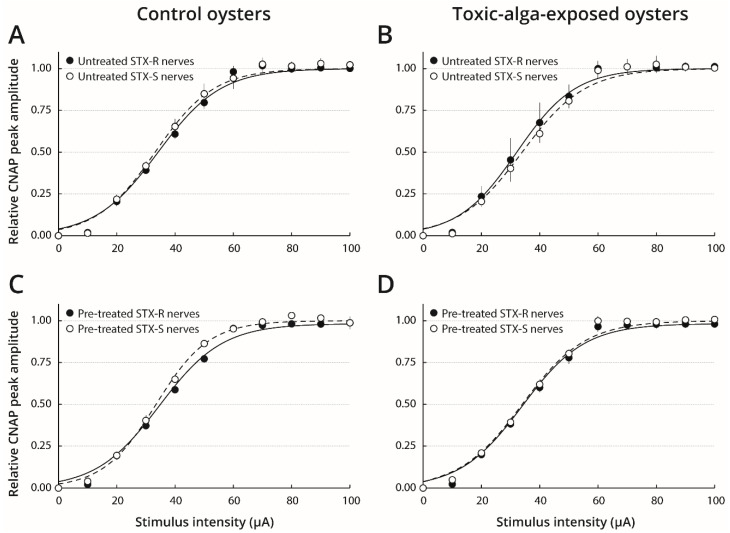
Response-intensity relationships of nerves isolated from farmed oysters. Response-intensity relationships of relatively sensitive (STX-S, ○) and relatively resistant (STX-R, ●) nerves isolated from farmed oysters fed with *T. lutea* (control oysters, **A**,**C**) or *A. minutum* (toxic-alga-exposed oysters, **B**,**D**), before (untreated, **A**,**B**) and after (pre-treated, **C**,**D**) their pre-treatment with 8.39 µM STX. CNAP peak amplitudes (means ± SE of 6–12 nerves) were expressed relatively to their respective maximal amplitude, as a function of the intensity of 1-ms duration stimulus. The curves correspond to data fits according to the Boltzmann equation with the I_50_ and S values indicated in Table 3.

The membrane excitability level of STX-sensitive and STX-resistant nerves isolated from toxic-alga-exposed and control oysters was also analysed by establishing response-intensity relationships and determining the corresponding variables, before and after nerve pre-treatment with 8.39 µM STX (Figure 4 and Table 3). Comparison of response-intensity relationships of untreated-nerves isolated from either toxic-alga-exposed or control oysters revealed no significant difference in response-intensity relationships, I_50_ and S values between STX-sensitive and STX-resistant nerves. Similar results were obtained after pre-treatment of nerves with 8.39 µM STX. Moreover, the response-intensity relationships also were similar, and corresponding variables were not statistically different after toxin pre-treatment of either STX-sensitive or STX-resistant nerves when compared with those of untreated nerves, regardless of whether the oysters were fed with toxic or non-toxic microalgae. These results indicate that the membrane excitability characteristics of nerves were not a distinguished criterion of their sensitive or resistance character to the toxin.

### 2.4. TTX Sensitivity of Oyster Nerves

The results show that none of the nerves isolated from oysters collected on October 2015 was detected to be TTX-sensitive. In particular, CNAP maximal peak amplitude measured after pre-treatment of nerves with 7.85 µM TTX, and expressed as a percentage of the value determined before pre-treatment, was 96 ± 11% (*n* = 7). Similar results were obtained with nerves isolated from oysters sampled on May 2016 and exposed to toxic (PST-producing) or non-toxic microalgae (see Materials and Methods): relative CNAP maximal peak amplitudes, determined after nerve pre-treatment with 7.85 µM TTX, were 91 ± 14% (*n* = 12) and 96 ± 15% (*n* = 11), respectively.

### 2.5. Relation Between STX Bio-Accumulation in Digestive Glands and Sensitivity of Nerves

**Figure 5:**
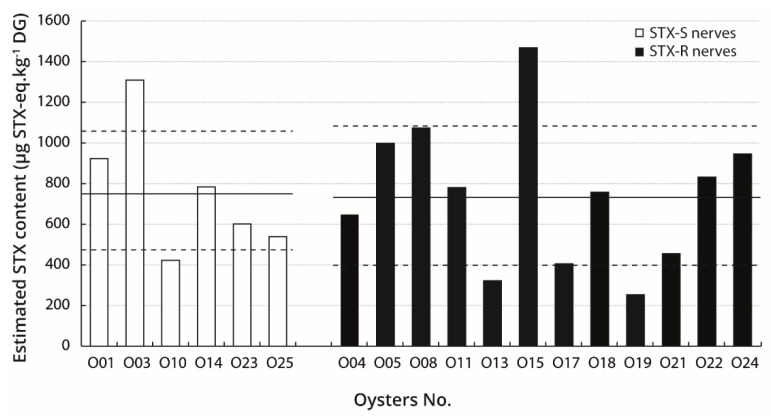
Individual levels of bio-accumulated PST in farmed oysters fed with *A. minutum* (toxic-alga-exposed oysters). Histogram of individual estimated STX contents in digestive glands of toxic-alga-exposed oysters possessing STX-sensitive (STX-S) nerves (□, *n* = 6) and STX-resistant (STX-R) nerves (■, *n* = 12). In each case, full and dashed lines indicate mean and SE values, respectively, i.e., 760 ± 290 µg STX-eq.kg^−1^ DG (□) and 740 ± 340 µg STX-eq.kg^−1^ DG (■).

The STX contents in individuals were quantified in digestive glands of oysters exposed to toxic and non-toxic microalgae. Results are as follows: 750 ± 320 µg STX-eq.kg^−1^ DG for toxic-alga-exposed oysters (*n* = 18) and no PST was detected in the digestive glands of control oysters (*n* = 10). The estimated STX contents in the digestive glands of toxic-alga-exposed oysters were analysed with respect to the STX sensitivity of the nerves. No significant difference in the mean estimated STX content was detected between oysters possessing STX-sensitive nerves, i.e., 760 ± 290 µg STX-eq.kg^−1^ DG (*n* = 6), and those having STX-resistant nerves, i.e., 740 ± 340 µg STX-eq.kg^−1^ DG (*n* = 12) (Figure 5). Moreover, no statistically significant correlation was observed between the estimated STX contents and any of the CNAP variables in oysters possessing either STX-sensitive or STX-resistant nerves.

## 3. Discussion

The present electrophysiological evaluation of STX and TTX sensitivities of cerebrovisceral nerves isolated from *C. gigas* reveals that (1) CNAP maximal peak amplitude was found to be ~0.42 mV and to propagate at a velocity of 2.6 m/s under control conditions, (2) CNAP maximal peak amplitude was inhibited by micromolar-range concentrations of STX, whereas similar TTX concentrations were ineffective, (3) two categories of oyster nerves could be distinguished according to relative STX sensitivity, (4) STX content in oysters did not appear correlated to nerve sensitivity to STX, and (5) the STX sensitivity of nerves was lower when oysters were previously exposed to the PST-producing *A. minutum* dinoflagellate, compared with those fed with a non-toxic microalga.

### 3.1. Cerebrovisceral Nerves of Pacific Oysters Are Suitable for Electrophysiological and Pharmacological Studies

The cerebrovisceral nerve of *C. gigas* was dissected with relative ease and showed good survivability (50% of nerves survived for 24 h after dissection), which makes this nerve a suitable model to study the electrophysiological and pharmacological properties of shellfish unmyelinated axons. Our results reveal that the CNAP maximal peak amplitude of the *C. gigas* cerebrovisceral nerve was consistently found to be ~0.42 mV and to propagate at a velocity of 2.6 m/s under control conditions. These values are of the same magnitude as previously reported for the cerebrovisceral nerve dissected from other bivalves, such as the freshwater mussels *Anodonta cataracta* and *Elliptio complanata*, the marine mussel *Mytilus edulis* [13], and the butter clam *Saxidomus giganteus* [15].

### 3.2. Cerebrovisceral Nerves of Pacific Oysters Have Intermediate STX Sensitivity Compared to Other Bivalves

In our study, STX acted on *C. gigas* nerves by decreasing the peak amplitude of individual action potentials of fibres without any marked change in membrane-excitability characteristics. Partial inhibition of CNAP was observed with micromolar STX concentrations, indicating that the sensitivity of *C. gigas* nerves to STX is intermediate among bivalve species. This sensitivity appears similar to that of two other bivalves, *Tresus capax* and *Protothaca staminea*, species with reported CNAP partial inhibition by micromolar STX concentrations [15]. In contrast, in other bivalves such as *S. giganteus* and *Saxidomus nuttalli*, millimolar STX concentrations were necessary to fully block CNAP, suggesting a higher resistance to this toxin than in *C. gigas* nerves [13]. In *Crassostrea virginica*, full blockage was observed with sub-micromolar STX concentrations, indicating that *C. virginica* nerves are more sensitive to the toxin than those of *C. gigas*. Several other studies demonstrated that the resistance characteristics of nerve fibres to STX and/or TTX differ between species. Indeed, STX and TTX resistance of nerves was reported in pufferfishes, crabs, newts and molluscs [13,14,16,17]. Additionally, micromolar TTX concentrations had no effect on CNAP of *C. gigas* nerves, indicating that these nerves are resistant to TTX. Discrepancy between the sensitivity of nerves to STX and TTX has been observed for several bivalve species including *M. arenaria* and *S. nuttalli* [10].

### 3.3. STX Sensitivity of Cerebrovisceral Nerves of C. gigas Oysters Varies Between Individuals

The nerves isolated from wild oysters collected on April 2014 were, on average, ~5 and ~20 fold more sensitive to STX than those of wild and farmed oysters collected on October 2015 and May 2016, respectively. This variability could reflect a seasonal variability or can originate from their growing conditions. For example, wild oysters are cemented on rock, whereas farmed oysters are free in bags. These hypotheses need to be tested by further experiments performed monthly on oysters from different geographical origin and life conditions. The observed variability could also result from inter-individual variability in nerve sensitivity. Indeed, studying the STX sensitivity of toxic-alga-exposed and control oyster nerves reveals that, at the highest concentration tested, the toxin partially inhibited the CNAP of some nerves while being ineffective on others. This prompts to postulate on the existence of sensitive and resistant categories of nerves in Pacific oysters. These two categories of nerves may originate from differential expression of alternative splice transcripts of Na_v_ channels (CgNav1) reported to be expressed in *C. gigas* nerves [11], which may have different electrophysiological and pharmacological properties. Indeed, in leeches and jellyfishes, electrophysiological studies showed two types of sodium currents with different kinetics and STX sensitivity [18,19]. This hypothesis needs to be tested by recording the STX sensitivity of each splice variant of oyster Na_v_ channels using, for example, heterologous expression in *Xenopus* oocytes, before a definitive conclusion can be reached. If, as expected, different STX sensitivities will be detected, it is likely that they will originate in conformational modifications of the protein and not in amino acid mutation(s) since the protein sequence of the PST binding site has been reported to appear perfectly conserved in all the individuals analysed [11]. It is worth noting that the presence of a glutamine residue in the PST binding site (Domain II) was hypothesised to be responsible for the intermediate sensitivity of *C. gigas* nerves to STX (see above) [11]. The relative resistance of *C. gigas* nerves to STX could also be explained if the CNAP is not generated by an increase in Na conductance but by an increase in Ca conductance. However, Twarog et al. [13] observed that the CNAP of some bivalve species (including *Crassostrea virginica*) was reduced and blocked, within minutes, by Na-deficient and Na-free solutions, considering thus unlikely that relative resistance to STX results from a CNAP generating mechanism independent of Na ions.

Whether the inter-individual variability of nerve sensitivity to STX may explain, or not, that of PST load in oysters is questionable.

### 3.4. The Variability of PST Load in Oysters Does Not Appear Related to Sensitivity of Nerves

In general, STX-resistant bivalve species bio-accumulate higher levels of toxins than STX-sensitive species [10,13], but our results show that this relationship appears to be more complex when considering intra-specific variability. The estimated STX contents in the digestive glands of toxic-alga-exposed Pacific oysters ranged from 250 to 1470 µg STX-eq.kg^−1^ DG and showed substantial inter-individual variability (up to five-fold). However, no correlation was found between the STX sensitivity of nerves and the estimated STX contents in digestive glands. It is likely that other variables, such as filtration activity and toxin assimilation, are involved in PST bio-accumulation in bivalve molluscs. Additionally, the present results show that *C. gigas* nerves are resistant to TTX, whereas recent studies point out controversial results concerning this toxin and analogues bio-accumulation in oysters, even using optimized and in-house validated methods [20,21,22]. This further supports that the relationship between toxin load in bivalve molluscs and toxin-sensitivity of nerves is more complex than expected.

### 3.5. A Pre-Exposure of Pacific Oysters to PST-Producer Dinoflagellates Decreases STX Sensitivity of Nerves

The resistance of nerves to STX was consistently associated with CNAP having relatively small maximal peak amplitude, high half-width, and low maximal rise and decay slopes. The membrane-excitability characteristics of nerves are not distinguishing criteria. Based upon the percentages of STX-sensitive nerves and their IC_50_ values, our results strongly suggest that the nerves of oysters, recently exposed to *A. minutum*, are more resistant to STX than those of control oysters. This could potentially correspond to a phenomenon of acclimation or desensitization and may be, once again, correlated to the relative expression of alternative splice transcripts of Na_v_ channels [11]. This phenomenon, potentially temporary and reversible, has to be distinguished from the genetic resistance resulting from natural selection on Na_v_ gene, as observed in *M. arenaria* softshell clam populations having a long-term history of PST exposure in the field [9]. As a result, the recent life history of oysters, in particular that related to neurotoxin exposure, can modulate the nervous sensitivity to PST and subsequent physiological responses and, indirectly, PST bio-accumulation. This result is of particular interest knowing that STX-producing *A. minutum* blooms often occur over several weeks during the spring–summer period in the Bay of Brest, an important zone for oyster production [23].

In conclusion, C. gigas nerves are shown, for the first time, to have micromolar range of STX sensitivity which decreases, at least temporary, upon recent oyster exposure to dinoflagellates producing PST under natural, but not experimental environment. Additionally, this work highlights the good potential of cerebrovisceral nerves of Pacific oysters for electrophysiological and pharmacological studies.

## 4. Materials and Methods

STX and TTX sensitivity of cerebrovisceral nerves of oysters was evaluated using an electrophysiological approach. Some oysters were exposed experimentally to a toxic strain of A. minutum. In parallel, the STX contents in oyster digestive glands were estimated using an ELISA assay.

### 4.1. Oysters

A first set of two preliminary experiments was performed on wild Pacific oysters, *C. gigas*, collected on rocks on April 2014 and October 2015 in the Bay of Brest (Le Dellec, Brittany, France). Oysters were kept in tanks filled with aerated seawater for a few days, prior to electrophysiological experiments. A second set of experiments was carried out on Pacific oysters obtained on May 2016 from a shellfish farm in the Bay of Brest (Logonna-Daoulas, Brittany, France). These oysters were experimentally exposed to toxic and non-toxic microalgae (see below).

### 4.2. Microalgal Cultures

The dinoflagellate *A. minutum* Halim (1960) strain Daoulas 1257 (isolated in Brittany, France), known to produce PST [24], was used for toxic algal exposure of oysters, and *Tisochrysis lutea* was used for non-toxic algal exposure of oysters, as control. The dinoflagellate *A. minutum* was cultured in L1 medium [25] at 16 °C, with a light/dark cycle of 12/12 h, and was harvested during the exponential growth phase. *T. lutea* microalga was grown in Conway medium [26]. Algal cell densities were determined by counts using Nageotte or Malassez cells under a light microscope.

### 4.3. Experimental Design for Exposure to Toxic and Non-Toxic Microalgae

On May 2016, oysters were randomly distributed in 18-L tanks. Each tank was supplied with microalgae using a peristaltic pump, and central air-lifts were used to mix microalgal cells continually. Oysters were fed during 4 days with a continuous seawater flow of 4.3 L/day containing either A. minutum [16.10^3^ cells/mL, which is in the range of a natural bloom concentration [23], *n* = 30 toxic-alga-exposed oysters, i.e., 3 replicates of 10 individuals] or T. lutea (4.10^5^ cells/mL, *n* = 23 control oysters, i.e., 3 replicates of 7–8 individuals). At the end of the exposure period (end-point phase), oyster digestive glands were dissected, weighed, frozen, and stored in liquid nitrogen until biochemical analyses. Nerves were isolated and immediately tested or put in standard physiological solution until being tested.

### 4.4. ELISA Assay for Estimated STX Content analyses

Digestive glands were ground with an “ultraturax” homogenizer and extracted in 0.1 M HCl (1:1, w:w). The mixture was boiled at 104 °C for 10 min and then cooled to room temperature. After centrifugation (3500× *g*, 10 min, 4 °C), supernatants were collected for analysing toxin content. The toxin content was assessed with the Saxitoxin (PSP) ELISA, Microtiter Plate kit (Abraxis, Warminster, PA, USA), according to manufacturer instructions, and expressed in µg STX-eq.kg^−1^ of digestive gland (DG) wet weight.

### 4.5. Electrophysiological Recordings

**Figure 6:**
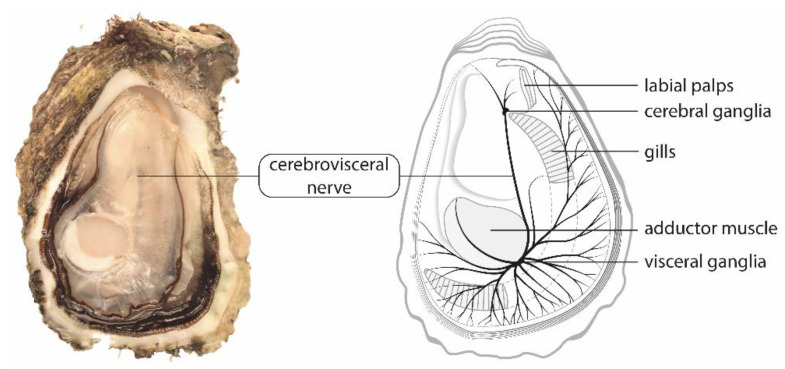
The cerebrovisceral nerves of the Pacific oyster, *C. gigas*, in the left valve. This pair of nerves, which rises from the posterior side of cerebral ganglia to the anterior side of visceral ganglia, is embedded in the connective tissue of the visceral mass. The visceral ganglia, located on the anteroventral side of the adductor muscle, at the junction between smooth and striated muscles, sends nerves to the adductor muscle, the posterior part of the mantle, the gills, the heart, the gonad and the digestive gland.

Electrophysiological recordings were performed on the cerebrovisceral nerves isolated from oysters. This pair of nerves originates from the cerebral ganglia and extends to the visceral ganglia through the connective tissue of the visceral mass (Figure 6). This renders the dissection of non-stretching nerves rather difficult. The dissected nerves were approximately 1 mm in diameter and at least 2 cm in length and could be obtained in about 10 min. When isolated, the nerves were either tested directly for functional state (see below) or kept in standard physiological solution (436 mM NaCl, 10 mM KCl, 52 mM MgCl_2_, 10 mM CaCl_2_ and 10 mM HEPES, pH adjusted to 7.39 with NaOH) until being tested. This solution approximates the molarities of major ions present in the chemical composition of salinity-35 sea water, with the exception of HEPES that is included to avoid pH changes that may affect the electrophysiological responses of nerves.

Electrophysiological recordings were performed at room temperature (20–22 °C) using a conventional electrophysiological technique (Supplementary Figure S4A), as previously described [27]. Briefly, the cerebrovisceral nerve was placed on five uninsulated platinum wires fixed in a moist Plexiglas chamber. Two wires (stimulating electrodes) were connected to a digital-analogic converter (Axon™ Digidata® 1440A Digitizer, Molecular Devices, Sunnyvale, California, USA) through which the computer delivered, at a frequency of 5 Hz, a series of square-wave pulses of 0.1–1 ms duration increasing in intensity from 0 to 300 µA in 10-µA steps. Two other wires (recording electrodes) were connected to a high-gain differential input amplifier followed by a second amplifier (home-made amplifiers, Ray Kado, CNRS, Gif-sur-Yvette, France), to record the CNAP. The amplified signal of the second was digitalized by an analogic-digital converter and stored on the computer. Axon pCLAMP 10.5 hardware and software (Molecular Devices, Sunnyvale, CA, USA) were used to both stimulate the nerve and record the CNAP response. The fifth wire, located midway between the stimulating and recording pairs of wires, was connected to the ground. The nerve was neither bathed nor super-fused during the recording period (which lasted less than 5 min) to avoid shunting effects of the medium. The Plexiglas chamber was covered and the humidity inside was ensured by wads of cotton soaked with standard physiological solution. It is worth noting that no, or only very negligible, stimulus artefacts were detected during electrophysiological recording, likely due to the experimental conditions (filters, recording periods…) and to the highly sophisticated recording Plexiglas chamber, specifically made for simultaneous X-ray scattering and electrophysiological studies of pike olfactory CNAP [28]. Absence of stimulus artefacts was also observed when recording pike olfactory CNAP under similar conditions [27].

### 4.6. Toxins

Saxitoxin dihydrochloride (STX), reference NRC-CRM-STX provided by Novakits (Nantes, France), was prepared as a 66 µM stock solution in aqueous 3 mM hydrochloric acid, and then diluted in the standard physiological solution to give final concentrations of between 0.07 and 8.39 µM. The final concentration of hydrochloric acid in the STX medium did not exceed 0.38 mM. Tetrodotoxin citrate (TTX), purchased from Alomone Labs (Jerusalem, Israel), was prepared as a 1 mg/mL stock solution in distilled water and then diluted in the standard physiological solution to yield a final concentration of 0.06 or 7.83 µM. The nerve responsiveness was tested before and after being pre-treated by 20–30 min incubation in a given STX or TTX medium.

### 4.7. Data Analyses

To evaluate the membrane excitability level of nerves, response-intensity relationships were established by plotting the peak amplitude (A) of CNAP, expressed relative to maximal amplitude (A_max_) recorded in response to large stimulus intensities, as a function of stimulus intensity (I). The theoretical curves correspond to data point fits, according to the Boltzmann equation (GraphPad Prism 5 software): A/A_max_ = 1 − [1/(1 + exp((I − I_50_)/S))], where I_50_ is the stimulus intensity producing a CNAP with 50% maximal peak amplitude, and S is the slope of the curve.

To characterise the kinetics of a given CNAP, six variables were measured (Supplementary Figure S4B): the maximal peak amplitude (in mV), the width at 50% peak amplitude (half-width in ms), the area (in mV.ms), the time between stimulation onset and peak (time-to-peak in ms), the maximal rise slope (in mV/ms), and the maximal decay slope (in mV/ms). The velocity of CNAP propagation was approximated by calculating the ratio of the distance between stimulating and recording electrodes, i.e., 15 mm, and the time-to-peak.

To evaluate the nerve sensitivity to STX, concentration-response relationships were established by plotting the maximal peak amplitude of CNAP, recorded from STX-pre-treated nerves (A_maxS_), and expressed as percentage of the value obtained in absence of toxin (A_maxC_), against the STX concentration ([STX]). The theoretical curves were calculated from typical sigmoid nonlinear regressions through data points according to the Hill equation (GraphPad Prism 5 software): A_maxS_/A_maxC_ = 1/[1 + ([STX]/IC_50_)^nH^], where n_H_ is the Hill number and IC_50_ is the STX concentration necessary to inhibit 50% of CNAP maximal peak amplitude.

Data are presented as the mean ± standard error (SE) of *n* nerves. Statistical analyses were executed with R 3.2.2 (R Core Team, 2015). Comparison of data between different experimental conditions applied to the same oyster group of nerves was performed with a paired Student’s *t*-test when normality and homoscedasticity were observed or, otherwise, with Wilcoxon signed rank test. Comparison of data between two different oyster groups of nerves subjected to the same experimental conditions was performed with Welch two sample *t*-test when normality and homoscedasticity were observed or, otherwise, with the Wilcoxon rank sum test.

## Figures and Tables

**Table 1:** CNAP variables determined from STX-untreated nerves of farmed oysters fed with *A. minutum* (toxic-alga-exposed oysters) or with *T. lutea* (control oysters).

	Maximal Peak Amplitude (mV)		Half-Width (ms)		Area (mV.ms)		Time-to-Peak (ms)		Maximal Rise Slope (mV/ms)		Maximal Decay Slope (mV/ms)	
	Before SPS	After SPS	Before SPS	After SPS	Before SPS	After SPS	Before SPS	After SPS	Before SPS	After SPS	Before SPS	After SPS
Control oysters (*n* = 21 nerves)	0.374 ± 0.097	0.358 ± 0.081	5.124 ± 0.794	5.025 ± 0.607	2.088 ± 0.336	1.995 ± 0.316	56.079 ± 0.396	55.995 ± 0.249	0.250 ± 0.090	0.252 ± 0.076	0.116 ± 0.047	0.105 ± 0.038
Toxic-alga-exposed oysters (*n* = 25 nerves)	0.269 ± 0.102 **	0.303 ± 0.095 *	6.201 ± 1.156 **	5.780 ± 1.026 **	1.822 ± 0.530	1.930 ± 0.405	56.364 ± 0.346 *	56.265 ± 0.308 **	0.179 ± 0.076 **	0.193 ± 0.084 *	0.075 ± 0.036 **	0.087 ± 0.047

SPS: standard physiological solution. * 0.01 < *p* < 0.05 and ** 0.001 < *p* < 0.01, compared with control oysters. See Supplementary Figure S4B for details.

**Table 2:** CNAP variables determined from STX-sensitive (STX-S) and STX-resistant (STX-R) nerves isolated from farmed oysters fed with *A. minutum* (toxic-alga-exposed oysters) or with *T. lutea* (control oysters), before STX pre-treatment of nerves.

	Maximal Peak Amplitude (mV)		Half-Width (ms)		Area (mV.ms)		Time-to-Peak (ms)		Maximal Rise Slope (mV/ms)		Maximal Decay Slope (mV/ms)	
	STX-S Nerves	STX-R Nerves	STX-S Nerves	STX-R Nerves	STX-S Nerves	STX-R Nerves	STX-S Nerves	STX-R Nerves	STX-S Nerves	STX-R Nerves	STX-S Nerves	STX-R Nerves
Control oysters (n = 8–10 nerves)	0.393 ± 0.051	0.317 ± 0.069 *	4.705 ± 0.387	5.294 ± 0.652 *	1.935 ± 0.292	1.923 ± 0.339	55.992 ± 0.301	56.004 ± 0.250	0.274 ± 0.068	0.176 ± 0.073 *	0.126 ± 0.030	0.086 ± 0.033 *
Toxic-alga-exposed oysters (n = 6–12 nerves)	0.387 ± 0.074	0.339 ± 0.083	5.154 ± 0.374	5.445 ± 0.915	1.891 ± 0.248	2.035 ± 0.377	56.389 ± 0.354	56.139 ± 0.308	0.215 ± 0.298	0.196 ± 0.038	0.133 ± 0.035	0.100 ± 0.050

* 0.01 < *p* < 0.05, STX-resistant *versus* SYX-sensitive nerves of control oysters.

**Table 3:** Variables of response-intensity relationships determined from STX-sensitive (STX-S) and STX-resistant (STX-R) nerves isolated from farmed oysters fed with *A. minutum* (toxic-alga-exposed oysters) or with *T. lutea* (control oysters), before (untreated) and after pre-treatment of nerves with 8.39 μM STX.

	I_50_ (μA)				S (μA^−1^)			
	Untreated STX-S	Pre-Treated STX-S	Untreated STX-R	Pre-Treated STX-R	Untreated STX-S	Pre-treated STX-S	Untreated STX-R	Pre-Treated STX-R
Control oysters (n = 8–10 nerves)	33.16 ± 0.95	33.47 ± 1.06	34.18 ± 0.51	34.59 ± 0.62	10.10 ± 0.59	9.09 ± 0.24	10.77 ± 0.33	10.81 ± 0.28
Toxic-alga-exposed oysters (n = 6–12 nerves)	33.97 ± 0.74	34.10 ± 0.82	32.12 ± 3.81	34.06 ± 0.97	10.82 ± 0.23	10.58 ± 0.18	10.04 ± 2.10	10.70 ± 0.27

I_50_, stimulus intensity producing a CNAP with 50% maximal peak amplitude; and S, slope of the curve.

## Data Availability

Data is contained within the article and supplementary materials.

## Supplementary Materials

The following are available online at https://www.mdpi.com/article/10.3390/md19070380/s1, Figure S1: comparison of compound nerve action potential (CNAP) recorded from nerves of farmed oysters fed with *A. minutum* (toxic-alga-exposed oysters) or *T. lutea* (control oysters), Figure S2: separation of nerves dissected from farmed oysters fed with *A. minutum* (toxic-alga-exposed oysters) or *T. lutea* (control oysters) according to their response to 8.39 µM STX, Figure S3: STX effects on relatively resistant nerves of farmed oysters fed with *A. minutum* (toxic-alga-exposed oysters) or *T. lutea* (control oysters), Figure S4: experimental set-up for recording oyster compound nerve action potential (A) and description of measured response variables (B).
