# Supplementary material for: Electrophysiological Evaluation of Pacific Oyster (Crassostrea gigas) Sensitivity to Saxitoxin and Tetrodotoxin
Source: Mar Drugs. 2021 Jun 30;19(7):380. doi: 10.3390/md19070380 (PMC8307532; doi:10.3390/md19070380)
Supplement: Supplementary file 1 [file marinedrugs-19-00380-s001.zip › marinedrugs-1251331-supplementary.pdf]

Electrophysiological evaluation of Pacific oyster (*Crassostrea gigas*) sensitivity to saxitoxin and tetrodotoxin

Floriane Boullot, Caroline Fabioux, Hélène Hégaret, Pierre Boudry, Philippe Soudant, and Evelyne Benoit

SUPPLEMENTARY FIGURE S1

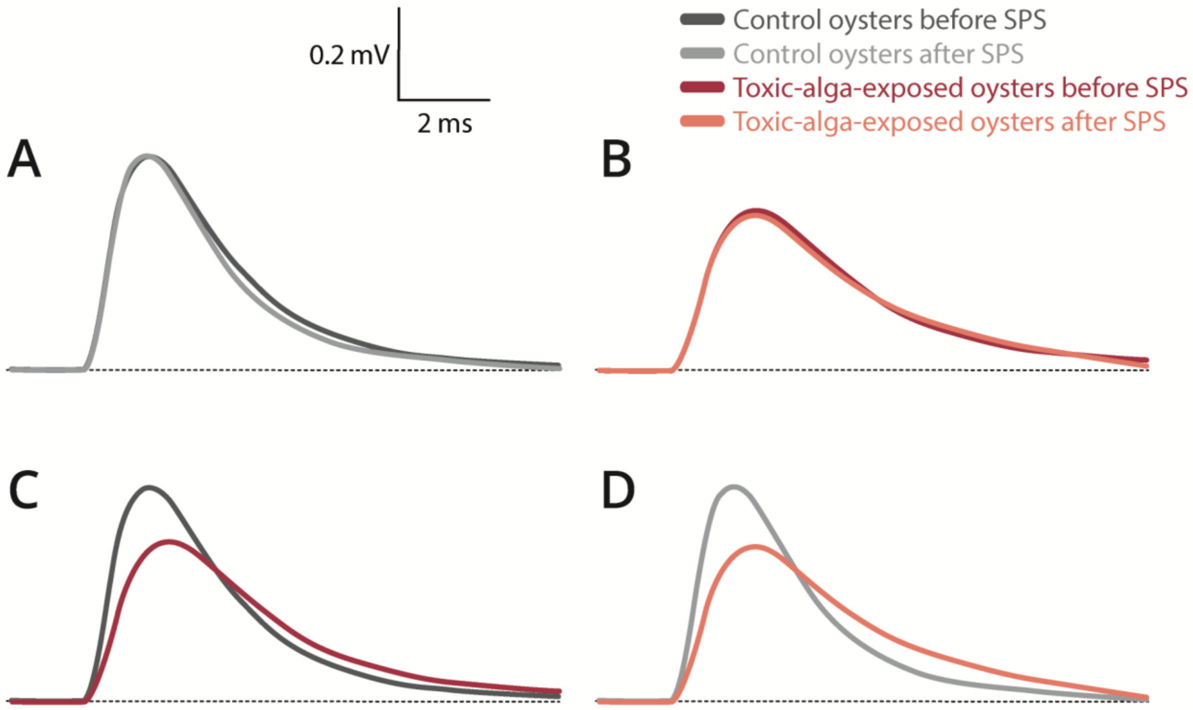

Comparison of compound nerve action potential (CNAP) recorded from nerves of farmed oysters fed with *A. minutum* (toxic-alga-exposed oysters) or *T. lutea* (control oysters). Superimposed traces of typical CNAP recorded in response to stimuli of 1 ms duration and 300 µA intensity from cerebrovisceral nerves isolated from toxic-alga-exposed (in red) and control (in black) oysters and submitted directly to a first recording session (dark colours) and then, kept in standard physiological solution (SPS) for less than 1 minute before undergoing a second recording session (light colours).

Electrophysiological evaluation of Pacific oyster (*Crassostrea gigas*) sensitivity to saxitoxin and tetrodotoxin

Floriane Boullot, Caroline Fabioux, Hélène Hégaret, Pierre Boudry, Philippe Soudant, and Evelyne Benoit

SUPPLEMENTARY FIGURE S2

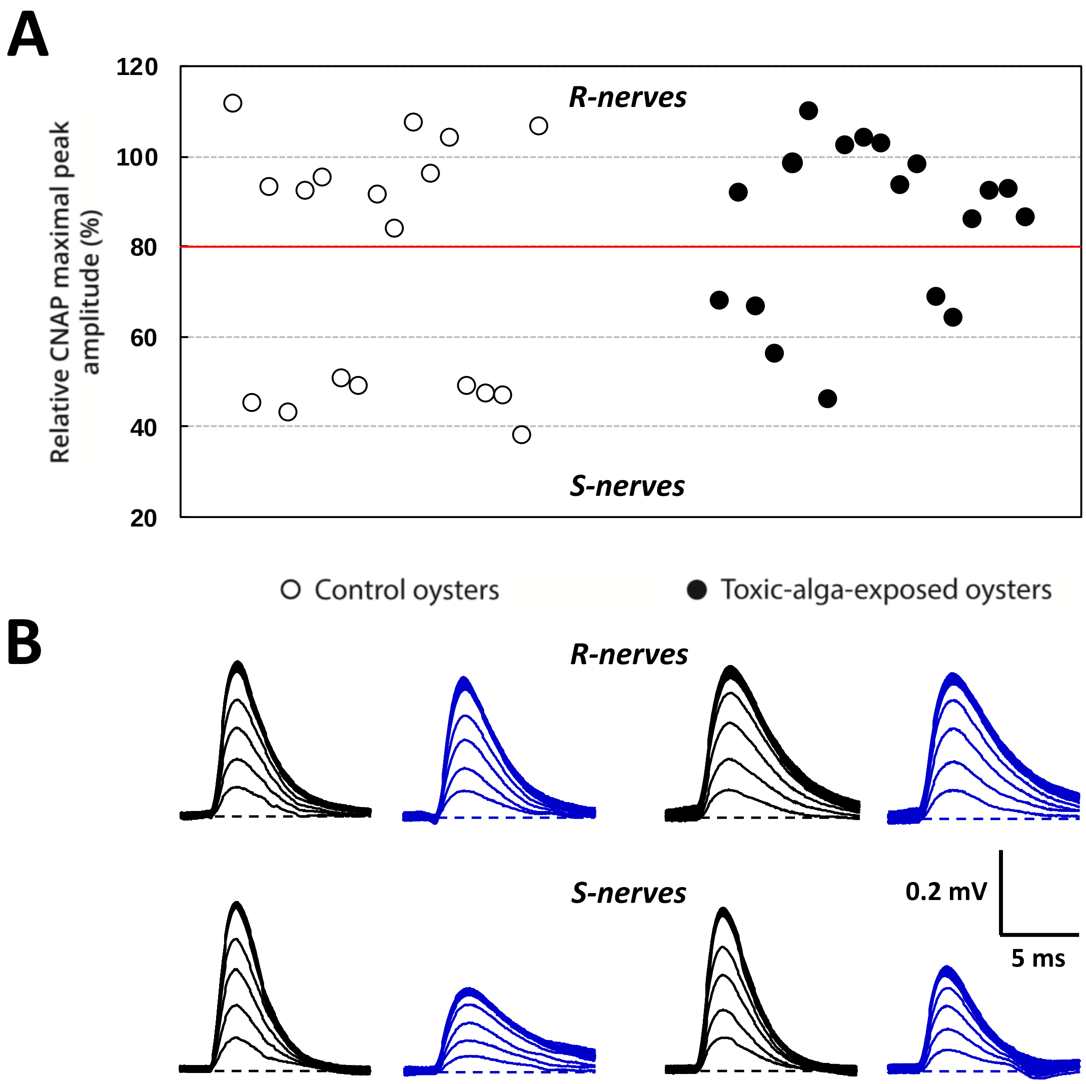

Separation of nerves dissected from farmed oysters fed with *A. minutum* (toxic-alga-exposed oysters) or *T. lutea* (control oysters) according to their response to 8.39 µM STX. (**A**) The compound nerve action potential (CNAP) was recorded from each toxic-alga-exposed (●) and control (○) oysters, before (in black) and after (in bleu) pre-treatment of nerves with 8.39 µM STX. (A) Maximal peak amplitudes (n = 18 nerves in each case) expressed as percentage of that obtained in absence of toxin. After pre-treatment of nerves with STX, the relative CNAP maximal peak amplitude of a given nerve was either higher than 84% or lower than 69%, leading to separate nerves into STX-resistant nerves (R-nerves, *i.e.* nerves with relative CNAP maximal peak amplitude higher than 80%) and STX-sensitive nerves (S-nerves, *i.e.* nerves with relative CNAP maximal peak amplitude lower than 80%). (**B**) Superimposed traces of typical CNAP recorded, under each condition, from the cerebrovisceral nerves stimulated with pulse intensities of 10-300 µA in 10-µA steps and 1-ms duration.

Electrophysiological evaluation of Pacific oyster (*Crassostrea gigas*) sensitivity to saxitoxin and tetrodotoxin

Floriane Boullot, Caroline Fabioux, Hélène Hégaret, Pierre Boudry, Philippe Soudant, and Evelyne Benoit

SUPPLEMENTARY FIGURE S3

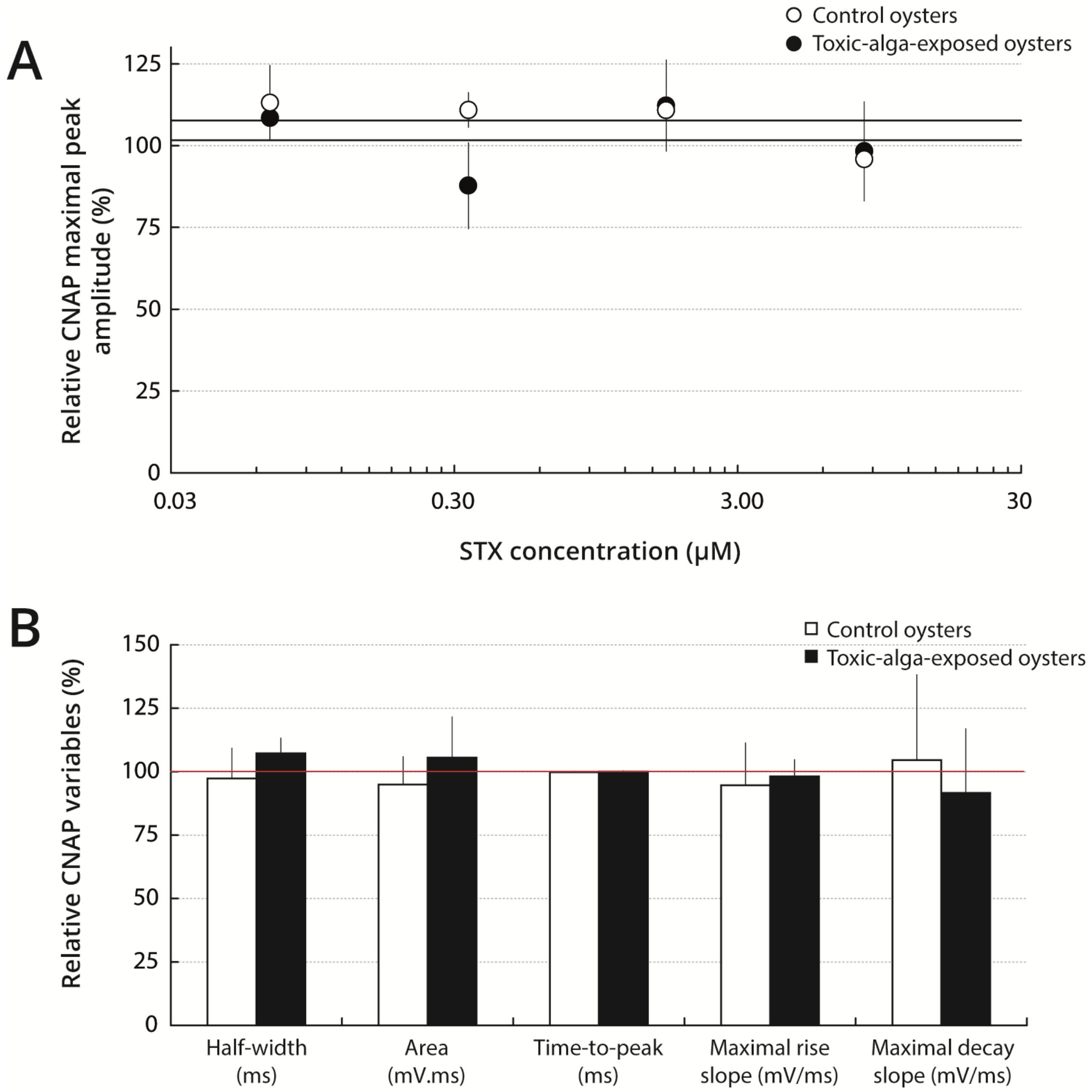

STX effects on relatively resistant nerves of farmed oysters fed with *A. minutum* (toxic-alga-exposed oysters) or *T. lutea* (control oysters). (**A**) Concentration-response curves of STX effects on the compound nerve action potential (CNAP) maximal peak amplitude recorded from toxic-alga-exposed (●) and control (○) oysters. Each value represents the mean ± SE of data obtained from 3-12 nerves, and is expressed as percentage of its value obtained in absence of toxin. The straight lines were calculated from linear regression through data points. (**B**) Histogram of variables characterising the CNAP kinetics of nerves isolated from toxic-alga-exposed (■) and control (□) oysters and pre-treated with 8.39 µM STX. Each value represents the mean ± SE of data obtained from 10-12 nerves, and is expressed as percentage of its value measured in absence of toxin.

Electrophysiological evaluation of Pacific oyster (*Crassostrea gigas*) sensitivity to saxitoxin and tetrodotoxin

Floriane Boullot, Caroline Fabioux, Hélène Hégaret, Pierre Boudry, Philippe Soudant, and Evelyne Benoit

SUPPLEMENTARY FIGURE S4

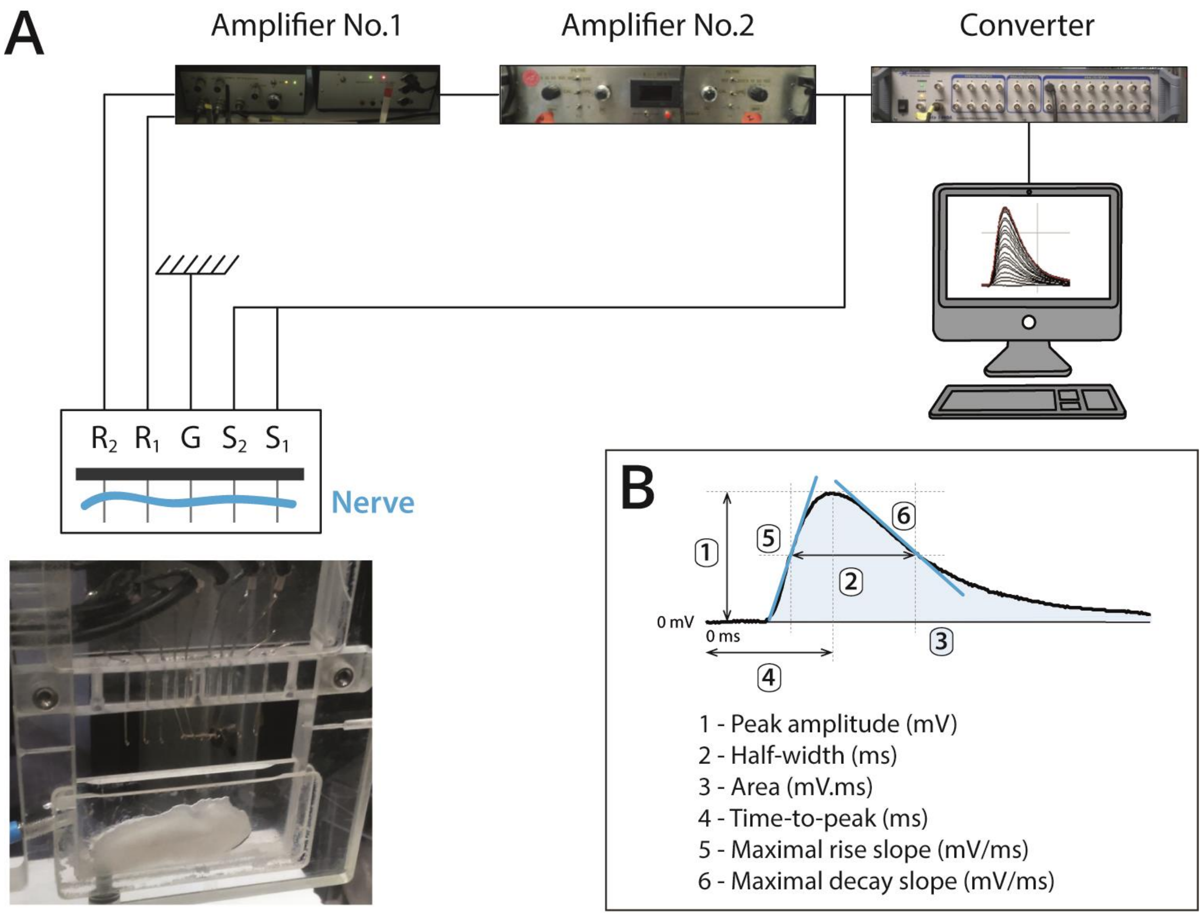

Experimental set-up for recording oyster compound nerve action potential (A) and description of measured response variables (B). (**A**) A given isolated cerebrovisceral nerve was placed on five uninsulated platinum wires fixed in a Plexiglas chamber. The stimulating electrodes (S_1 and S_2) were connected to a digital-analogic converter through which the computer delivered square-wave pulses. The recording electrodes (R_1 and R_2) were connected to a first high-gain differential input amplifier and then to a second one, to record the compound nerve action potential (CNAP). The amplified signal of the second was digitalized, through the analogic-digital converter, and stored on the computer. An electrode, located midway between the stimulating and recording pairs of electrodes, was connected to the ground. The Plexiglas chamber was covered and the humidity inside was ensured by wads of cotton soaked with standard physiological solution. (**B**) Six variables (indicated in inset) were measured to characterize the CNAP recorded under various experimental conditions.
